# Role of plasma Von-Willebrand factor in children with chronic liver diseases

**DOI:** 10.1007/s00431-025-06355-7

**Published:** 2025-08-05

**Authors:** Ola Galal Ali Behairy, Abdelhamid Mohamed El-Hamshary, Ahmed Adel Torky, Rana Atef Khashaba, Basma Galal Ali, Nashwa Farouk Mohamed

**Affiliations:** 1 Pediatrics Department, Faculty of Medicine, Benha University, Benha, Egypt; 2 M.B.B.Ch, Faculty of Medicine, Cairo University, Menoufia, Cairo, 32861 Egypt; 3 Clinical Pathology Department, Faculty of Medicine, Benha University, Benha, Egypt

**Keywords:** Von Willebrand factor, Children, Chronic liver diseases, Fibrosis

## Abstract

**C**hildren with chronic liver diseases (CLD) face more significant clinical problems. The most reliable technique for detecting liver fibrosis is still liver biopsy (LB). Our investigation assessed Von-Willebrand Factor Antigen (VWF Ag) plasma titer as a probable non-invasive predictor for grading liver fibrosis in children with CLD. 120 children participated in our case–control study, 60 of whom had CLD and the remaining 60 of whom were healthy. Underlying etiologies were diagnosed via clinical, biochemical, and histological criteria by LB. VWF Ag concentrations were determined in all participants using enzyme-linked immunosorbent assay (ELISA). VWF Ag mean titers were significantly elevated in CLD cases in contrast with controls (581.4 ± 279 vs. 166.9 ± 78 ng/ml; *P* < 0.001). There was a remarkable positive association amongst VWF Ag and total, direct serum bilirubin (TSB, DSB), aspartate aminotransferase (AST), alanine aminotransferase (ALT), liver span, portal hypertension, Model for End-Stage Liver Disease (MELD) score, Pediatric End-Stage Liver Disease (PELD) score, and Child–Pugh score (*P* ≤ 0.05). VWF Ag anticipated mild (AUC 0.973, cutoff > 266.5 ng/ml) and severe fibrosis (AUC 0.988, cutoff > 590.3 ng/ml) with sensitivity and specificity of 100% and 86.7% for mild fibrosis and 100% and 92.9% for severe fibrosis, respectively.

*Conclusion*: VWF Ag can serve as a significant indicator of liver fibrosis severity in pediatric CLD cases.

**Table:** 

**What is Known:** • *Liver biopsy remains the cornerstone for diagnosing and staging liver fibrosis in children*.
**What is New:** • *VWF Ag is a promising non-invasive indicator for grading liver fibrosis severity in pediatric chronic liver disease*. • *VWF Ag correlates strongly with fibrosis scores as Child–Pugh, MELD, PELD, AST/PLT ratio index (APRI), and fibrosis-4 (FIB-4), and histological fibrosis stages (by ISHAK score) in children*.

**What is Known:**

• *Liver biopsy remains the cornerstone for diagnosing and staging liver fibrosis in children*.

**What is New:**

• *VWF Ag is a promising non-invasive indicator for grading liver fibrosis severity in pediatric chronic liver disease*.

• *VWF Ag correlates strongly with fibrosis scores as Child–Pugh, MELD, PELD, AST/PLT ratio index (APRI), and fibrosis-4 (FIB-4), and histological fibrosis stages (by ISHAK score) in children*.

## Introduction

Chronic liver disorder is defined by its duration (usually > 3–6 months) or by the presence of severe liver disease or physical features of chronic liver disease (clubbing, jaundice, or hepatosplenomegaly). The severity varies; the affected child may have simply biochemical indications of liver malfunction, findings of chronic liver disease, or manifest as hepatic failure. It is distinguished by the ongoing destruction and restoration of the liver parenchyma [1]***.*** CLD poses a significant global health issue, contributing to roughly 2 million deaths annually worldwide [2]**.**

Children's liver disease has different causes depending on their age. Neonates and infants are often present with biliary atresia, idiopathic neonatal hepatitis, sepsis, total parenteral nutrition-induced or genetic cholestatic syndromes (e.g., progressive familial intrahepatic cholestasis) [3]. Older children are more likely to have glycogen storage diseases, Alagille syndrome, Wilson disease, or viral hepatitis. Autosomal recessive polycystic kidney disease and other ciliopathies are linked to liver fibrosis in early childhood. Autoimmune hepatitis and Alpha-1-antitrypsin deficiency may be presented at any age [4].

Multiple cellular processes are involved in liver fibrosis. Hepatic cells respond to injury by releasing specific cytokines that promote inflammation and activate hepatic stellate cells into collagen-producing myofibroblasts, according to current models for liver fibrosis [5]. The majority of chronic liver illnesses result in the increasing buildup of fibrous tissue inside the liver, which ultimately causes hepatic insufficiency, portal hypertension, and liver cirrhosis [6]**.**

It is believed that endothelial dysfunction has a significant contribution to the emergence of various human illnesses, such as liver cirrhosis [7]**.** In cirrhotic liver, decreased bioavailability of vasodilator (nitric oxide) results in impaired endothelium-dependent relaxation in the hepatic microcirculation. This leads to an increase in intrahepatic vascular resistance, which ultimately results in portal hypertension [8]**.**

Weibel-Palade bodies are where VWF is constitutively produced and amassed in endothelial cells [9]**.** In the setting of vascular injury, VWF, a giant multimeric glycoprotein, induces platelets to latch on to the confronted subendothelium and serves a fundamental function in maintaining coagulation factor VIII [10]**.**

The mainstay for diagnosing and treating pediatric liver illness is still liver biopsy (LB). The LB function has also developed into a predictive tool for a number of liver illnesses, offering details like the staging of fibrosis and histologic grades of inflammation. A tiny biopsy sample may reveal localized and misleading liver abnormalities in many pediatric illnesses [11]**.** Fever, hematoma, discomfort, and supraventricular tachycardia are the main side effects after LB [12]**.**

This research assessed the plasma VWF Ag titer as a simple predictor for detecting and categorizing the stage of liver fibrosis among children with CLD.

## Patients and methods

This case–control analysis comprised 60 children and adolescents (up to 18 years old) with CLD of various causes, selected from Benha University Hospital's Pediatric Department's Pediatric Hepatology Clinic (32 patients were males and 28 were females with a mean age ± SD of 9 ± 4 years). An additional 60 healthy children from the overall community who were matched by age and sex served as the control group (22 children were males and 38 were females with a mean age ± SD of 11 ± 2 years).

### Inclusion criteria for cases

Children under 18 years of both sexes were diagnosed with CLD, including hepatitis B or C, autoimmune hepatitis, cholestatic liver disorders, or metabolic liver disorders, with liver fibrosis previously identified through clinical, biochemical, and radiological assessments and confirmed by LB.

### Inclusion criteria for controls

Controls were healthy children who volunteered from outpatient clinics during routine check-ups for the purpose of sport training and school routine examination, matched for age/sex, with normal liver function investigations, with no previous history of a liver disorder.

### Exclusion criteria for both case and control

Malignancies, severe systemic diseases (active sepsis, advanced cardiopulmonary or renal dysfunction, connective tissue or neoplastic disorders), and hematologic conditions (e.g., von Willebrand disease, thrombotic thrombocytopenic purpura, bone marrow disorders, or possible hemolytic–uremic syndrome) were excluded.

### Sample size calculation

The sample size was calculated using G*power software version 3.1.9.7 based on a previous study [13], which reported a large effect size of Von Willebrand Factor between patients and controls. The total sample size needed to detect a large effect size (d = 0.8) of Von Willebrand Factor between the studied groups was 90 patients. The sample was increased to 120 patients (60 per group) to compensate for possible use of non-parametric tests and possible laboratory failure. Alpha and power were adjusted for 0.05 and 0.95, respectively.

**All participants were subjected to**

A. Clinical Evaluation

A thorough physical examination and history were taken. Body mass index (BMI), weight, and height were all assessed as part of anthropometrics. Egyptian pediatric references were used to compute percentiles.

B. Radiological investigations

All cases were asked to report abdominal ultrasound and Doppler for detection of ascites, portal hypertension, and size of liver and spleen.

C. Laboratory investigations

***A complete blood count (CBC)*** was made with 1 mL of whole blood collected in an EDTA (Ethylene-diamine-tetra-acetic acid) vacutainer and gently mixed to prevent clotting. Hematological parameters, including hemoglobin concentration (Hb), mean corpuscular volume, mean corpuscular hemoglobin, red cell distribution width, and mean corpuscular hemoglobin concentration, were measured using the Sysmex KX-21N automated hematology analyzer (Sysmex Corporation, New York, USA). For differential leukocyte counts, peripheral blood smears were prepared and stained using Leishman’s stain.

***Liver function tests*,** including aspartate aminotransferase (AST), alanine aminotransferase (ALT), alkaline phosphatase, gamma-glutamyl transferase (GGT), total serum protein, serum albumin, total serum bilirubin (TSB), and direct serum bilirubin (DSB), were performed following the collection of 4 mL of venous blood into plain tubes devoid of anticoagulant. Samples were given time to coagulate, then centrifuged at 1500 rpm for 15 min. The biochemical analyses were carried out using the Biosystem A1A autoanalyzer (Spain).

***Coagulation profiles***, including prothrombin time (PT), partial thromboplastin time (PTT), and international normalized ratio (INR), were evaluated using the HUMACLOT DUE PLUS® coagulation analyzer (Wiesbaden®, Germany).

In cases diagnosed with ***autoimmune hepatitis***, gamma globulin titer, anti-nuclear antibodies (ANA), anti-smooth muscle antibody (ASMA), and anti–liver-kidney microsomal (anti-LKM) antibody titers were determined using the indirect immunofluorescence technique with INOVA Diagnostics, Inc. (Germany), offering NOVA Lite Rat Stomach, Kidney, and Liver kits.

***Plasma VWF antigen (VWF Ag) titer*** was measured using the Human vWF ELISA Kit (Catalog No: DLR-vWF-Hu). Samples of peripheral blood were drawn without the use of a tourniquet, placed in tubes with heparin or EDTA as anticoagulants, and brought to the lab in an iced box. Samples were centrifuged at 1000 × g for 15 min at 2–8 °C within 30 min of assembly. For further examination, the extracted plasma was aliquoted and kept at −20 °C or −80 °C. VWF Ag typically ranges between 50 and 200 ng/ml.

***Non-invasive serological markers*** were used for detection of fibrotic liver. These include APRI score (AST/platelet ratio index) and fibrosis-4 (FIB-4) score using age, platelet count, ALT and AST.

D. Liver biopsy (LB) and staging

LB was done for CLD patients only. Every patient had a liver biopsy performed using the Menghini aspiration needle method under ultrasound guidance. All biopsies contained ≥ 11 portal tracts (median: 15, ranged from 11 to 20) as confirmed by histopathology. Formalin and paraffin were used to preserve biopsy specimens, which were then placed on a glass slide and stained with hematoxylin and eosin to determine the histological activity of hepatitis using the Ishak score. Masson trichrome was used to determine the stage of fibrosis. The fibrosis stages extended from F0 to F6 (F0 = no fibrosis, F1 = mild, F2-3 = moderate, F4-6 = severe) [14]**.** Patients with cirrhosis were categorized clinically according to Child–Pugh, MELD, and PELD scores as follows:

- ***PELD score:*** For younger children (below 12 years old), it was used to evaluate its impact on growth and decide whether liver transplantation was necessary. It is useful for estimating the predicted death rate from hepatic disease. It was calculated according to the formula; PELD = 4.80[Ln serum bilirubin (mg/dL)] + 18.57[Ln INR]—6.87[Ln albumin (g/dL)] + 4.36 (< 1 year old) + 6.67(growth failure) [15]***.***
- ***MELD score:*** It proved to be helpful in assessing prognosis and setting priorities for liver transplant recipients. It was calculated according to the following formula: MELD = 3.78 [Ln serum bilirubin (mg/dL)] + 11.2 [Ln INR] + 9.57 [Ln serum creatinine (mg/dL)] + 6.43 [16]***.***
- ***Child–Pugh scoring system:*** Its purpose was to forecast mortality in cirrhosis patients. Patients were divided into three groups: those with excellent liver function (A), those with mild liver function impairment (B), and those with advanced liver dysfunction (C). To classify patients, they used five clinical and laboratory criteria: prothrombin time, ascites, neurological disorders, serum bilirubin, and serum albumin [17, 18]***.***

### Statistical analysis

Data were analyzed using SPSS v28. Normality was assessed via the Shapiro–Wilk test and visual inspection. Normally distributed data were reported as mean ± SD, non-normal as median (range), and categorical variables as n (%). Group comparisons used t-test/Mann–Whitney U (quantitative) or chi-square/Fisher’s exact (categorical). Kruskal–Wallis test with Dunn’s post-hoc correction was used for ≥ 3 non-parametric groups. ROC analysis evaluated VWF’s diagnostic performance for liver fibrosis (area under the curve, AUC, cutoff, sensitivity/specificity). Spearman’s correlation assessed relationships. Significance was set at p < 0.05 [19].

## Results

This study involved 120 children, categorized into two groups: the 1 st one comprised 60 hepatic cases diagnosed with CLD, while the 2nd one included 60 healthy children serving as controls. A statistically significant increase in positive consanguinity and family history of liver diseases was observed among hepatic cases in contrast with the control group. In terms of anthropometric data, hepatic cases showed significantly lower height percentile, weight, weight percentile, and BMI percentile in comparison to controls. Both groups had comparable age and sex **(**Table 1**).**

**Table 1:** Demographic, anthropometric and etiological distributions of the studied groups

Variables		Hepatic Cases (*n* = 60)	Controls (*n* = 60)	*P*-value
**Age (years)**	Mean ± SD	9 ± 4	11 ± 2	0.19
	Median (min–max)	7.5 (0.2–17)	9.5 (0.5–17)	0.09
**Sex**				
Males	n (%)	32 (53)	22 (37)	0.14
Females	n (%)	28(47)	38 (63)	
Positive Consanguinity	n (%)	42 (70)	3 (5)	< 0.001*
FH of liver diseases	n (%)	13 (21.7)	0 (0)	< 0.001*
Weight (KG)	Mean ± SD	25.1 ± 10.1	31.7 ± 14.9	0.005*
Weight (percentile)	Median (min–max)	10 (1—77)	32 (1—84)	< 0.001*
Height (cm)	Mean ± SD	120.2 ± 26.4	127.3 ± 28.8	0.16
Height (percentile)	Median (min–max)	6 (1—93)	12 (1—98)	0.002*
BMI	Mean ± SD	15.1 ± 3	17.6 ± 4.1	< 0.001*
BMI (percentile)	Median (min–max)	19 (1—65)	58 (1—98)	< 0.001*
Etiology of hepatic cases				
GSDs	n (%)	39 (65%)		
Type Ia		13 (22%)		
Type Ib		9 (15%)		
Type III		15 (25%)		
Type IX		2 (3%)		
Auto immune hepatitis		14 (23%)		
Congenital hepatic fibrosis		3 (5%)		
Idiopathic neonatal hepatitis		3 (5%)		
Dorfman syndrome		1 (2%)		

^*^Significant *P*-value, *SD* Standard deviation, *n* Number, %: Percentage, *FH* Family history, *KG* Kilograms, *cm* Centimeters, *BMI* Body mass index, *min* minimum, *max* maximum, *GSDs* Glycogen storage diseases

The clinical presentations of hepatic cases reveal that most individuals exhibited abdominal distension and pain (85%), while jaundice was observed in 60% of cases and fatigue in 30%. A small percentage of cases presenting with convulsions was reported in 5% of the cases and bleeding (in the form of hematemesis and melena) in 3%. Also, it is noted that the age of onset of liver disease ranged from 0.2 to 16 years old, with an average age of 4.3 years, and the duration of liver disease ranged from 0.4 to 13.5 years, with a mean of 5.14 years. All cases were presented with hepatomegaly, with liver span ranging from 7.5 to 19 cm, and 32% of cases presented with splenomegaly, with spleen size ranging from 6 to 12.5 cm.

Glycogen storage disease (GSDs) is the most common diagnosis, affecting 39 cases (65%) of which there were 13 cases with Type Ia, 9 cases with Type Ib, 15 cases with Type III and 2 cases with Type IX. Autoimmune hepatitis (AIH) is detected in 23% of cases. Congenital hepatic fibrosis and idiopathic neonatal hepatitis each account for 5% of the diagnoses. Dorfman syndrome is identified in one case only **(**Table 1**).**

Hepatic cases demonstrated statistically elevated levels of TSB, DSB, AST, ALT, ALP, GGT, and total IgG in contrast with controls (*p* < 0.001). PT was slightly prolonged in the hepatic group. In contrast, Hb, albumin, and total serum protein titers were significantly lower in hepatic cases. No statistically significant differences were observed between the two groups regarding INR, PTT, white blood cell count (WBC), or platelet count (PLT) (*p* > 0.05). Additionally, 13% of hepatic cases tested positive for ASMA, while 10% were positive for ANA **(**Table 2**).**

**Table 2:** Laboratory findings in the studied groups

Variables		Hepatic Cases (*n* = 60)	Controls (*n* = 60)	*P*-value
TSB (mg/dl)	Median (min–max)	2.1 (0.3—17.6)	0.8 (0.4—1)	< 0.001*
DSB (mg/dl)	Median (min–max)	0.3 (0.1—4.6)	0.2 (0.1—0.3)	< 0.001*
AST (U/l)	Median (min–max)	150 (55—401)	28 (16—39)	< 0.001*
ALT (U/l)	Median (min–max)	89 (61—410)	21 (12—25)	< 0.001*
ALK ph. (U/l)	Mean ± SD	572 ± 122	162 ± 38	< 0.001*
GGT (U/l)	Median (min–max)	47 (22—520)	24 (12—45)	< 0.001*
PT (seconds)	Mean ± SD	14.1 ± 1.6	13.5 ± 0.9	0.011*
INR	Mean ± SD	1.3 ± 0.1	1.1 ± 0.1	0.180
PTT (seconds)	Mean ± SD	33 ± 5	32 ± 4	0.182
Total Serum protein (g/dl)	Mean ± SD	4.9 ± 0.5	5.9 ± 0.4	< 0.001*
Albumin (g/dl)	Mean ± SD	3.2 ± 0.3	3.8 ± 0.2	< 0.001*
	Median (min–max)	3.1 (2.5—3.6)	3.7 (3.4–3.9)	
Hb (g/dl)	Mean ± SD	10.4 ± 0.9	12.4 ± 0.6	0.010*
	Median (min–max)	9.2 (7.1–11.2)	11.4 (10.3–13.5)	
WBC (10^3/ul)	Mean ± SD	7.9 ± 3	7.4 ± 2	0.267
	Median (min–max)	6.2 (4.5–8.9)	5.8 (4.1–9.3)	
PLT (10^3/ul)	Mean ± SD	240 ± 118	278 ± 94	0.05
	Median (min–max)	199 (49–374)	205 (154–406)	
Total IgG (mg/dl)	Mean ± SD	1118 ± 377	980 ± 127	0.009*
	Median (min–max)	1212 (750–2016)	995 (640–1310)	
Positive ASMA	N (%)	8 (13)		
Positive Anti-Mitochondrial Abs	N (%)	0		
Positive ANA	N (%)	6 (10)		
Positive Anti-LKM Abs	N (%)	0		
plasma VWF titer (ng/ml)	Mean ± SD	581.4 ± 279	166.9 ± 78	< 0.001*
	Median (min–max)	511.6(285.5—1190.1)	142.3(55.4—395.3)	

^*^Significant *P*-value, *TSB*: Total serum bilirubin, *DSB* Direct serum bilirubin, *AST* Aspartate aminotransferase, *ALT* Alanine aminotransferase, *ALK ph.* Alkaline phosphatase, *GGT* Gamma-glutamyl transferase, *PT* Prothrombin time, *INR* International normalized ratio, *PTT* Partial thromboplastin time, *Hb* Hemoglobin, *WBC* White blood cell count, *PLT* Platelet count, *IgG* Immunoglobulin G. *n* Number, *%* Percentage, *ASMA* Anti-smooth muscle antibody, *Abs* Antibodies, *LKM* Liver kidney microsomal, *ANA* Antinuclear antibody, *VWF* von Willebrand factor, *min* minimum, *max* maximum

In hepatic cases, the degree of fibrosis is predominantly mild, affecting 70% of hepatic cases, with moderate fibrosis observed in 20% and severe fibrosis in 10%. Regarding histological activity, 65% of cases exhibit mild activity, 25% have minimal activity, and 10% have moderate activity. Also, the mononuclear inflammatory cells are the most prevalent, observed in 80% of hepatic cases. Lymphocytes are present in 20%, and plasma cells are identified in 15% of the cases, while eosinophils and multinucleated giant cells are each found in 10% of the cases **(**Table 3**).**

**Table 3:** Liver biopsy among the studied cases according to the Ishak scoring system

Variables			n	%
Degree of Fibrosis	Mild fibrosis	1–2/6	42	70
	Moderate fibrosis	3–4/6	12	20
	Severe fibrosis	5–6/6	6	10
Histological activity index	Minimal	1–3/18	15	25
	Mild	4–8/18	39	65
	Moderate	9–12/18	6	10
	Severe	13–18/18	0	0
Types of cells	Mononuclear Inflammatory Cells		48	80
	Lymphocytes		12	20
	Eosinophils		6	10
	Multinucleated giant cells		6	10
	Plasma Cells		9	15

*n* Number, % Percentage

**Fig. 1:**
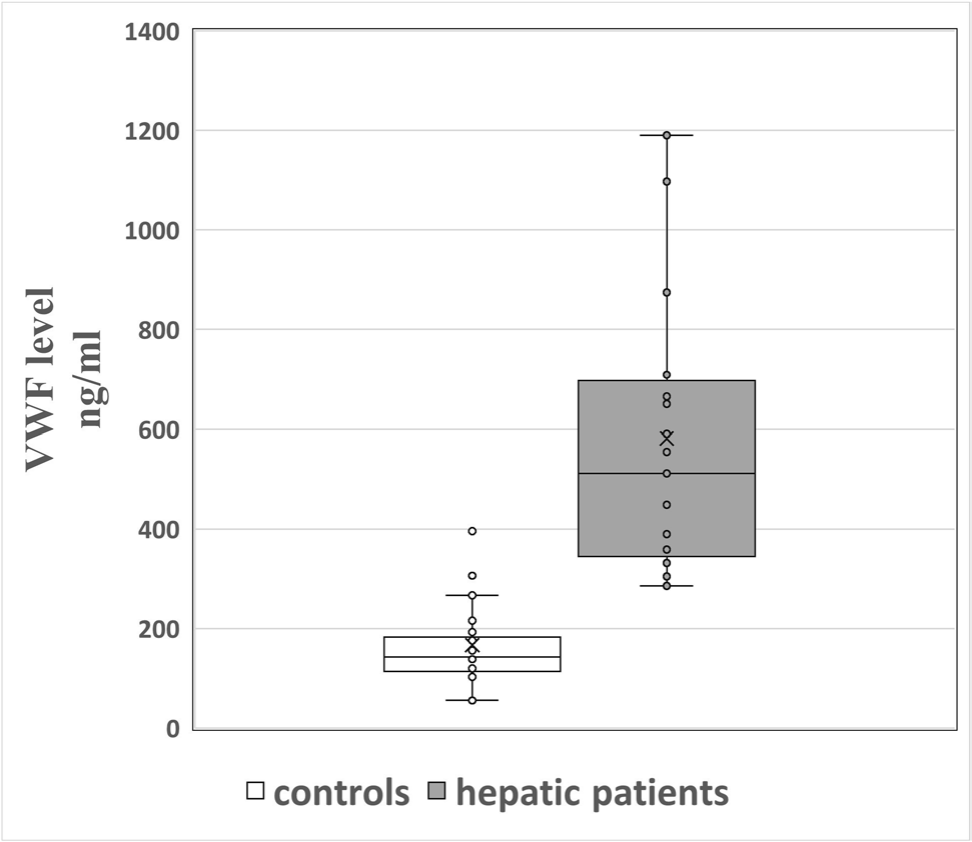
Boxplot showing the distribution of plasma VWF Ag levels (ng/ml) in CLD cases (*n* = 60) and controls (*n* = 60). The central line represents the median, the box spans the interquartile range (IQR; 25th–75th percentiles), and whiskers extend to the minimum and maximum values. Outliers are plotted as individual points

Plasma VWF titers were statistically increased in hepatic cases with a median of 511.6 ng/ml (range 285.5–1190.1) in contrast with 142.3 ng/ml (range 55.4–395.3) in controls (*P* < 0.001) **(**Fig. 1**) (**Table 2**).**

Plasma VWF titers have statistically positive correlations with TSB, DSB, AST, ALT, liver span, portal hypertension, MELD, PELD, Child–Pugh scores, APRI (AST to Platelet Ratio Index), and FIB-4 (Fibrosis-4) scores (*P* < 0.05). Furthermore, there is a negative correlation between plasma VWF titer and BMI, Hb, WBC, and PLT (*P* < 0.05). Other variables, including age, weight, height, ALP, PT, INR, PTT, total serum protein, albumin, total IgG, and ASMA, did not show statistical correlations with plasma VWF titer (*P* > 0.05) **(**Table 4**).**

**Table 4:** Correlation between plasma VWF and other parameters in hepatic cases

Variables	plasma VWF level	
	r	*P*
Age (years)	0.024	0.854
Weight (KG)	−0.103	0.432
Height (cm)	0.021	0.873
BMI	−0.383**	0.003*
TSB	0.390	0.002*
DSB	0.458	< 0.001*
AST	0.658	0.002*
ALT	0.765	0.003*
ALK ph	0.008	0.955
GGT	−0.093	0.478
PT	0.033	0.801
INR	−0.192	0.141
PTT	−0.198	0.128
Total serum protein	−0.023	0.861
Albumin	−0.126	0.337
Hb	−0.276	0.033*
WBC	−0.285	0.027*
PLT	−0.308	0.017*
Total IgG	−0.06	0.648
PELD score	0.303*	0.036*
MELD score	0.417	0.021*
Child–Pugh score	0.297	0.046*
APRI	0.420	0.002*
FIB-4	0.380	0.006*
ASMA	0.135	0.183
Liver span	0.680	< 0.001*
Portal hypertension	0.579	0.002*

^*^Significant *P*-value, *VWF* von Willebrand factor, *r* Correlation coefficient, *P* P-value, *KG* Kilograms, *cm* Centimeters, *BMI* Body mass index, *TSB* Total serum bilirubin, *DSB* Direct serum bilirubin, *AST* Aspartate aminotransferase, *ALT* Alanine aminotransferase, *ALK ph* Alkaline phosphatase, *GGT* Gamma-glutamyl transferase, *PT* Prothrombin time, *INR* International normalized ratio, *PTT* Partial thromboplastin time, *Hb* Hemoglobin, *WBC* White blood cell count, *PLT* Platelet count, *IgG* Immunoglobulin, *G PELD* Pediatric end-stage liver disease, *MELD* Model for end-stage liver disease, *ASMA* Autoimmune specific marker antibody, *APRI* AST To Platelet Ratio Index, *FIB* Fibrosis

Plasma VWF titer was statistically elevated in hepatic cases with advanced degrees of fibrosis and elevated histological activity index. Also, cases with mononuclear inflammatory cells and lymphocytes have a statistically elevated median VWF titer of 831.2 ng/ml in contrast with those without mononuclear inflammatory cells and lymphocytes, who have a median of 425.1 ng/ml. Plasma cells are associated with an elevated median VWF titer of 650.8 ng/ml in contrast with 448.3 ng/ml in cases without plasma cells. Also, according to the many causes of liver fibrosis, the plasma titer of VWF in hepatic patients does not exhibit statistical significance (p > 0.05) **(**Table 5**).**

**Table 5:** Plasma VWF according to results of liver biopsy and etiology of CLD

Variables			VWF titer (ng/ml)	*P*- value
Degree of Fibrosis	Mild	Mean ± SD	498 ± 99	< 0.001*
	Moderate		562 ± 102	
	Severe		603 ± 92	
Histological Activity Index	Minimal	Mean ± SD	401 ± 95	< 0.001*
	Mild		491 ± 87	
	Moderate		532 ± 101	
	Severe		0	
Type Of Cells	**Mononuclear Inflammatory Cells**			
	Yes	Median (min–max)	831.2 (511.6–1190.1)	< 0.001*
	No		425.1 (285.5–1097)	
	**Lymphocytes**			
	Yes	Median (min–max)	831.2 (511.6–1190.1)	< 0.001*
	No		425.1 (285.5–1097)	
	**Eosinophils**			
	Yes	Median (min–max)	533.3 (511.6–554.9)	0.674
	No		479.9 (285.5–1190.1)	
	**Multinucleated giant cells**			
	Yes	Median (min–max)	533.3 (511.6–554.9)	0.674
	No		479.9 (285.5–1190.1)	
	**Plasma Cells**			
	Yes	Median (min–max)	650.8 (590.3–1097)	0.012*
	No		448.3 (285.5–1190.1)	
Etiology of CLD	Autoimmune Hepatitis	Median (min–max)	650.8 (590.3—1097)	0.92
		Mean ± SD	779.4 ± 239	
	Congenital Hepatic Fibrosis	Median (min–max)	654.1 (552.1–861.6)	0.92
		Mean ± SD	689.2 ± 152.7	
	Glycogen Storage Disease	Median (min–max)	395.9 (285.5–1190.1)	0.078
		Mean ± SD	536.8 ± 298	
	Idiopathic Neonatal Hepatitis	Median (min–max)	621.3 (511.2–699.5)	0.92
		Mean ± SD	610.7 ± 123.3	

**Fig. 2:**
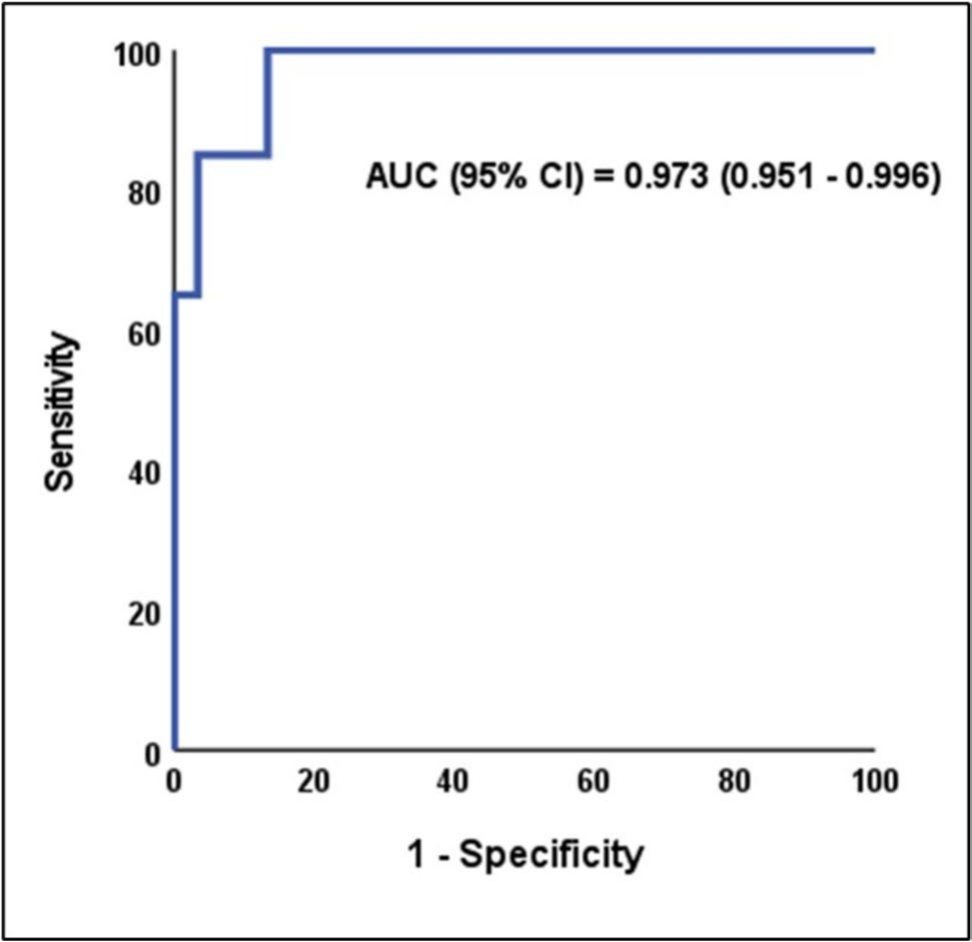
ROC analysis of plasma VWF to predict mild fibrosis

**Fig. 3:**
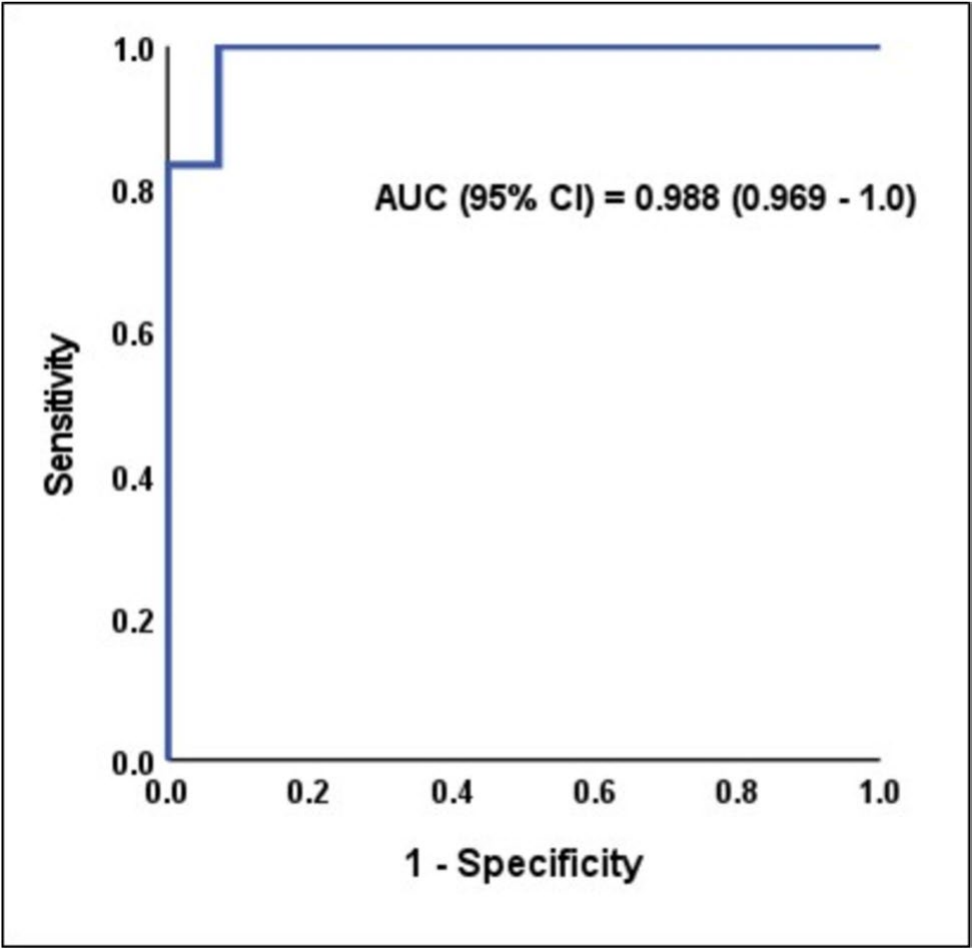
ROC analysis of plasma VWF to predict severe fibrosis

The ROC analysis for plasma VWF in predicting mild (F1-2) and severe (F5-6) liver fibrosis yields a statistically significant and excellent AUC, with a 95% confidence interval. The best cutoff value is > 266.5 ng/ml for mild fibrosis and > 590.3 ng/ml for severe fibrosis, at which sensitivity (100%), specificity (86.7%), positive predictive value (PPV) (88.2%), and negative predictive value (NPV) (100%) for mild fibrosis and sensitivity (100%), specificity (92.9%), PPV (85.7%), and NPV (100%) for severe fibrosis **(**Table 6**) (**Figs. 2 & 3**).**

**Table 6:** ROC analysis of plasma VWF to predict mild and severe liver fibrosis in CLD from controls

ROC characteristics	Mild fibrosis (F1-2)	severe fibrosis (F5-6)
AUC	0.973	0.988
95% CI	0.951–0.996	0.969–1.0
Best cutoff	> 266.5	> 590.3
Sensitivity	100%	100%
Specificity	86.7%	92.9%
PPV	88.2%	85.7%
NPV	100%	100%
P-value	< 0.001*	< 0.001*

^*^Significant *P*-value, *ROC* Receiver operating characteristic, *AUC* Area under the curve, *CI* Confidence interval, *PPV* Positive predictive value, *NPV* Negative predictive value

## Discussion

Liver fibrosis is a severe condition resulting from persistent hepatic damage caused by various liver diseases, including viral hepatitis, metabolic liver diseases, autoimmune hepatitis, and hereditary disorders. It significantly increases liver-related mortality, regardless of the cause, and can also affect liver transplant outcomes in children [20]**.**

GSDs emerged as the predominant diagnosis (65%), followed by AIH (23%), while congenital hepatic fibrosis and idiopathic neonatal hepatitis account only for 5% of cases. The high prevalence of glycogen storage disease reflects our center’s role as a referral site for metabolic disorders. Viral hepatitis was rare (0%), likely due to Egypt’s successful hepatitis C elimination program, while consanguinity (70% in cases) may increase autosomal recessive GSD.

VWF is a multimeric glycoprotein with adhesive properties, physiologically secreted by activated endothelial cells during the initial stages of hemostasis [21].

Notably, GSDs had lower median VWF levels (395.9 ng/ml) than AIH (650.8 ng/ml), possibly due to differing fibrogenic mechanisms. In metabolic diseases, fibrosis arises from hepatocyte accumulation, whereas autoimmune hepatitis drives endothelial activation via inflammation.

The current investigation was intended to determine if VWF Ag titer could identify and classify the degree of liver fibrosis in children with CLD.

When comparing hepatic cases to controls, the mean values of plasma VWF increased in a highly statistically notable manner (581.4 ± 279 vs. 166.9 ± 78) *(P* < *0.001)***.** Additionally, there were negligible variations in VWF Ag titer with respect to the cause of liver illness *(P* > *0.05)*. Furthermore, it was shown that those with extensive fibrosis had considerably higher VWF Ag levels *(P* < *0.05)*. Moreover, hepatic individuals with portal hypertension and esophageal varices had a considerably higher VWF Ag titer than those without these conditions **(*P* < 0.05).**

***Abdelrazek *et al*.'s*** [13] recent study in Egypt found that cirrhotic patients had a much higher mean VWF titer (167.1 ± 47.8 IU/dL) than healthy controls (112.9 ± 36.1 IU/dL) (P < 0.001). Both comparing cases to controls and stratifying cases by disease severity revealed this considerable increase: those with advanced fibrosis and varices had significantly higher VWF antigen titers (*P* < 0.05). Furthermore, strong statistical associations between VWF Ag titer and Child–Pugh score and fibrosis stage were found (*P* < 0.001). Higher VWF Ag levels in our study may reflect pediatric-specific pathophysiology or methodological variations (e.g., ELISA kit sensitivity).

The rise in VWF titer during liver fibrosis has been attributed to several mechanisms, including endothelial damage induced by bacterial components that enhance VWF secretion [22]**.** Cases with liver cirrhosis have been observed to have elevated VWF titers. Elevated VWF titer in cirrhosis may result from endotoxemia due to bacterial translocation and shear stress induced by hyperdynamic splanchnic circulation (portal hypertension), both of which may promote conformational changes in VWF that enhance its affinity for platelet binding [21]**.**

These results are aligned with those of ***Goel *et al*.*** [23], who demonstrated the role of VWF:Ag and its glycoprotein Ib binding activity as non-invasive biomarkers in pediatric portal hypertension (PHT). Elevated VWF:Ag was significantly associated with clinically significant varices (223 IU/dl; *p* = 0.015) and variceal bleeding (174 IU/dl; *p* = 0.077), indicating their potential as predictors of PHT-related complications. Notably, our study confirms these findings, as VWF Ag correlated strongly with PHT (r = 0.579, *P* = 0.002) and fibrosis severity **(**Table 4**)**, suggesting broader utility in pediatric CLD staging.

The current study's results demonstrate that VWF Ag is a major indicator of liver fibrosis (*P* < 0.001), demonstrating strong diagnostic performance across all evaluated cutoff values (> 266.5 in mild fibrosis and > 590.3 in severe fibrosis) with 100% sensitivity, specificity ranging from 86.7% to 92.9%, PPV ranging from 85.7% to 88.2%, and NPV 100%. The ROC analysis further validated plasma VWF as an excellent discriminator for both mild and severe fibrosis, with AUC values of 0.973 and 0.988, respectively. According to these results, plasma VWF is believed to be a promising simple indicator of the degree of fibrosis.

Also, the positive correlations between VWF Ag and markers of liver injury (e.g., AST, ALT, bilirubin, liver and spleen size) and the negative correlations with BMI, hemoglobin, WBC, and platelets affirm its relevance as a marker of liver pathology.

VWF Ag should be used in combination with conventional biomarkers and/or criteria for the identification of severe liver fibrosis in order to increase the ability to identify liver fibrosis stages [24]**.**

***Maieron *et al*.*** [25] used AUROC analysis to compare VWF's performance to METAVIR scoring system for staging liver fibrosis in order to investigate the diagnostic value of VWF in evaluating liver fibrosis. VWF was shown to be one of the best indicators for differentiating between instances with fibrosis (F1–F4) and those without (F0), with an AUROC of 0.703. The authors came to the conclusion that, in situations of chronic hepatitis C infection, VWF offers a straightforward yet useful method for grading liver fibrosis and detecting undetectable cirrhosis.

Similarly, ***Takaya *et al*.*** [23] found that VWF is a potentially useful biomarker to anticipate the onset of HCC and recognize severe forms of liver fibrosis. The VWF Ag AUC for the diagnosis of advanced liver fibrosis phase was 0.721.

VWF Ag titer and the degree of liver disease, as indicated by fibrosis stage, as well as PELD, MELD, and Child–Pugh scores among the patients under investigation, were shown to be statistically considerably associated in the current study (*P* ≤ 0.05). According to ***Lisman *et al*.*** [22], the median VWF propeptide titer in the reference group was 89% (*p* < 0.001), but the titers were significantly elevated in Child A, B, and C (488%, 711%, and 735%, respectively) with cirrhosis. Additionally, they noted that VWF and Child classification had a favorable association.

Since VWF Ag is an acute-phase reactant, fibrosis or inflammation (such as viral or autoimmune hepatitis) might cause its levels to increase [26]. Given the strong link between VWF Ag titer and both APRI and FIB-4 (P < 0.05), which suggests fibrosis, this study concludes that it is most likely caused by fibrosis. Also, its correlation with high liver span and portal hypertension (p < 0.05) was often due to fibrosis.

## Conclusion

VWF Ag titer may be a useful predictive indicator in assessing the intensity of liver fibrosis in children with CLD, as evidenced by its positive linkages with liver function parameters, fibrosis grade, and histological activity index.

## Limitations

The reliability of the findings may be impacted by the somewhat small sample size. Additionally, differences in VWF Ag titer across ages may affect the precision of the diagnosis. It is challenging to evaluate how VWF Ag titers fluctuate over time in response to therapy or as disease progresses without longitudinal follow-up. VWF activity should be measured alongside VWF Ag to detect functional deficits in cirrhotic patients. As we were a tertiary center, metabolic liver diseases were more predominant.

## Data Availability

No datasets were generated or analysed during the current study.
